# An American Text-Book of Pathology

**Published:** 1902-04

**Authors:** 


AN AMERICAN TEXT-BOOK OF PATHOLOGY.—Edited by Ludvig Hektoen, M. D., Professor of Pathology, Rush Medical College, Chicago; and David Riesman, M. D., Professor of Clinical Medicine, Philadelphia Polyclinic. Handsome imperial octavo of 1245 pages, 443 illustrations, 66 of them in colors. Philadelphia and London: W. B. Saunders & Co. 1901. Cloth, $7.50; sheep or half Morocco, $8.50 net.

This work consists, in a thorough and complete exposition, of the essential facts and principles of general pathology and pathological anatomy. In our opinion, it is the best work of the kind which has yet appeared in English. The editors have shown wisdom in associating with themselves as colloborators only men who are already well and favorably known to the profession as writers and teachers. Among these are Victor C. Vaughn on "Intoxica-

Dr. Lott's Paper on "The Drug Habit: Its Treatment," was published in this journal for November, 1901. It was read at the Calvert meeting of the Brazos Valley Association. Since its publication Dr. Lott has received so many letters from physicians asking if he would receive such cases for treatment that he takes this method of announcing that he is prepared to and will receive and treat cases of opium, cocaine, chloral or whiskey addiction by the hyoscine treatment as described in the paper referred to. The method is well known to the readers of this journal. It is speedy and painless. Physicians who do not care to treat these cases, or who are not prepared to do so, are invited to correspond with him, and can send them to him with every assurance that they will be in good hands and will be treated strictly in accordance with ethical principles. They will be cared for in a private family residence, where Dr. Lott will personally treat them, and will be in the charge of skilled, trained and experienced nurses. Every comfort and convenience will be furnished, in the privacy of a home (not a sanitarium), including good family fare and cooking; privacy, baths, etc. Ladies will be received as well as men, and will have the services and constant attention of trained lady-nurses. Dr. Lott refers by permission to the editor of the Texas Medical Journal. Address Dr. M. K. Lott, Cameron, Texas.

tions," A. A. Stevens on "The Circulatory System," Joseph McFarland on "The Respiratory System," F. H. Montgomery on "The Skin," Wm. C. Carter, of the University of Texas, on "The General Pathology of Fever," and others. R.

